# Tubulointerstitial injury in proteinuric chronic kidney diseases

**DOI:** 10.3389/fmed.2024.1478697

**Published:** 2024-10-28

**Authors:** Monica Cortinovis, Norberto Perico, Giuseppe Remuzzi

**Affiliations:** Istituto di Ricerche Farmacologiche Mario Negri IRCCS, Bergamo, Italy

**Keywords:** proteinuria, tubulointerstitial injury, chronic kidney disease, tubule toxicity, fibrosis

## Abstract

Proteinuria is an independent risk factor for chronic kidney disease progression and cardiovascular diseases. Apart from its prognostic role, the load of proteins that pass across the disrupted glomerular capillary wall trigger multiple pathophysiologic processes. These include, among others, intratubular complement activation and excessive proximal tubular reabsorption of filtered proteins, especially albumin and albumin-bound free fatty acids, which can set off several pathways of cellular damage. The activation of these pathways can cause apoptosis of proximal tubular cells and paracrine effects that incite the development of interstitial inflammation and fibrosis, ultimately leading to irreversible kidney injury. In this review, we provide a comprehensive overview of the current understanding on the mechanisms underlying the tubular toxicity of ultrafiltered proteins in the setting of proteinuric chronic kidney diseases. The acquired knowledge is expected to be instrumental for the development of novel therapeutic classes of medications to be tested on top of standard of care with optimized renin-angiotensin-aldosterone blockade and sodium-glucose cotransporter-2 inhibition, in order to further improve the clinical outcomes of patients with proteinuric chronic kidney diseases.

## Introduction

Irrespective of its underlying cause, proteinuria is a strong and independent risk factor of kidney function decline and cardiovascular diseases (1, 2). Several *in vitro* and *in vivo* studies have reported that, besides having a prognostic value, proteins filtered through the damaged glomerular capillary barrier have toxic effects on the tubulointerstitium which directly contribute to kidney disease progression (3). Under physiologic conditions, the low amounts of large proteins that escape the glomerular filtration barrier, including albumin and albumin-bound free fatty acids, are almost entirely retrieved by the proximal tubule via endocytosis through the megalin-cubilin receptor complex (4, 5). Upon cell internalization, albumin undergoes lysosomal degradation into amino acids, which return to the circulation across the basolateral membrane (4, 5). Glomerular injury increases the filtered load of proteins, thereby enhancing proximal tubular protein reabsorption, which sets off a number of different pathways of cellular damage. The activation of these pathways, in turn, can cause apoptosis of proximal tubular cells and paracrine effects that promote the development of tubulointerstitial inflammation and fibrosis, eventually leading to irreversible kidney damage (6, 7). In this review, we examine the mechanisms of tubular toxicity of proteins filtered by the disrupted glomerular capillary barrier, the pathways leading to interstitial inflammation, and ending with fibrosis. Finally, we explore emerging therapeutic strategies aimed to unleash endogenous tubulointerstitial repair in the diseased kidney.

## Tubular toxicity of filtered proteins

**Figure 1:**
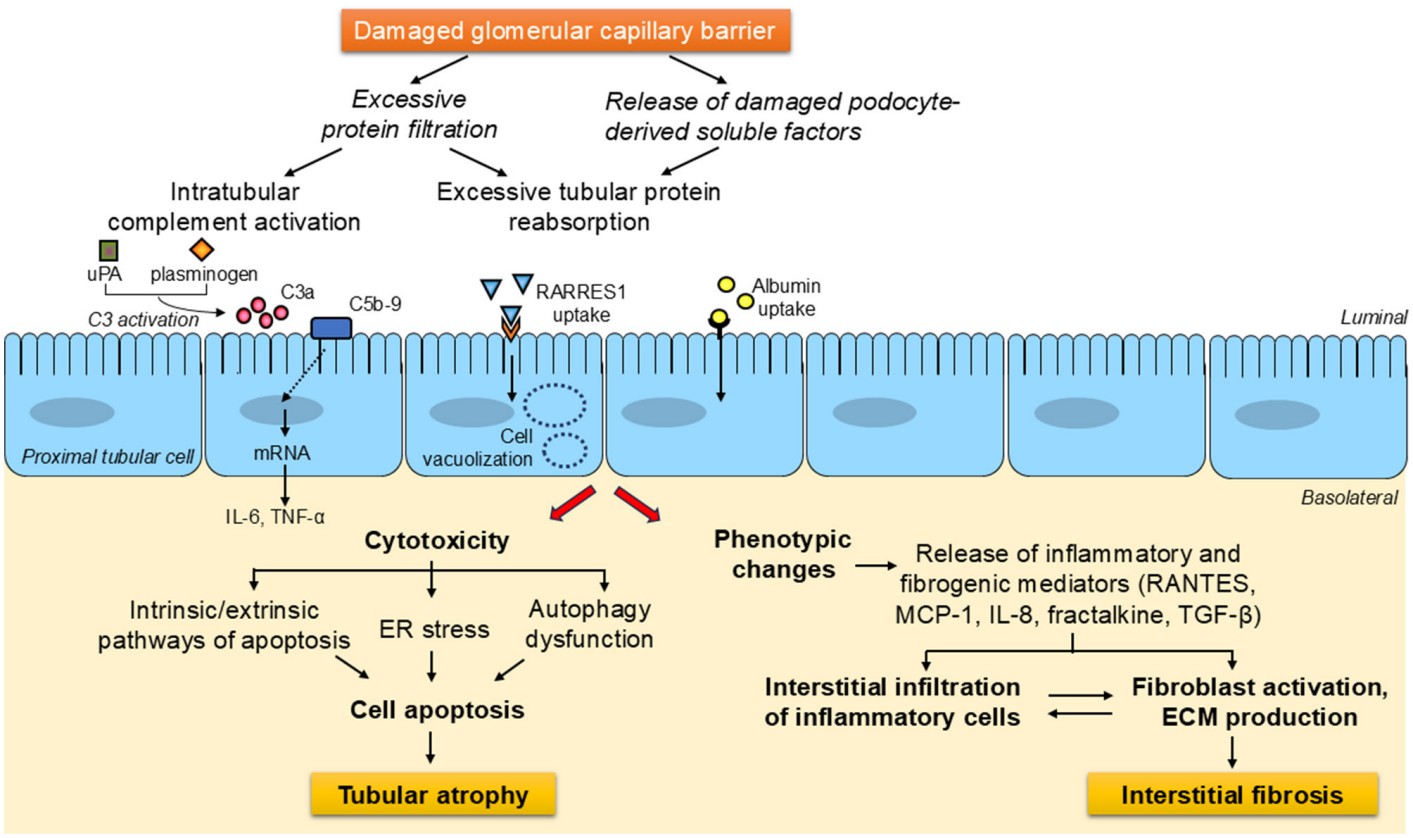
General overview of the signaling avenues and mechanisms downstream of damaged glomerular capillary barrier leading to tubulointerstitial injury in proteinuric chronic kidney diseases. Under proteinuric conditions, soluble factors cleaved from damaged podocytes and the load of proteins that escape the disrupted glomerular filtration barrier, including complement components and albumin, activate a wide array of pathways of tubular cell injury. These can lead to either cell apoptosis—through processes which can involve endoplasmic reticulum stress or autophagy impairment, eventually resulting in tubular atrophy -, or phenotypic changes. The latter generally entails the generation of inflammatory and fibrogenic substances, which are secreted into the interstitium, where they foster inflammatory cell infiltration and fibroblast activation, thereby contributing to the development of tubulointerstitial fibrosis. ECM, extracellular matrix; ER, endoplasmic reticulum; IL-6, interleukin-6; IL-8, interleukin-8; MCP-1, monocyte chemoattractant protein-1; RANTES, regulated upon activation, normal T cell expressed and secreted; RARRES1, retinoic acid receptor responder protein-1; TGF-β, transforming growth factor-β; TNF-α, tumor necrosis factor-α; uPA, urokinase-type plasminogen activator.

Under proteinuric conditions, soluble factors cleaved from damaged podocytes and excessive amounts of plasma-derived proteins that pass through the damaged glomerular filtration barrier, such as albumin and complement components, trigger the activation of multiple pathways of tubular cell injury, which result in either cell apoptosis—by mechanisms that can entail endoplasmic reticulum stress or autophagy dysfunction—or phenotypic changes. The latter mainly involves the production and secretion of pro-inflammatory and pro-fibrotic cytokines, both as soluble mediators and delivered via extracellular vesicles, which promote the recruitment of inflammatory cells and the activation of fibroblasts, ultimately leading to tubulointerstitial fibrosis (Figure 1).

### Cytotoxicity of damaged podocyte-derived soluble factors

Recent evidence suggests that, in response to injury, podocyte transmembrane proteins can undergo proteolytic cleavage into soluble factors which contribute to tubular damage. This appears to be the case for retinoic acid receptor responder protein-1 (RARRES1), a podocyte-enriched transmembrane protein whose expression is increased in kidney biopsies of patients with glomerular diseases (8). In mouse models of focal segmental glomerulosclerosis and diabetic kidney disease, podocyte-specific overexpression of RARRES1, but not of its cleavage mutant, exacerbated albuminuria, glomerular dysfunction, and tubular injury, pointing to soluble RARRES1 as the main factor responsible for the reported effects (9). Soluble RARRES1 cleaved from podocytes underwent endocytic uptake by proximal tubular cells *in vivo*, which associated with worsen tubular injury, as assessed by tubular vacuolation and kidney injury molecule-1 expression (9). Other experiments showed that the podocyte-specific matrix metalloprotease 23 was involved in RARRES1 cleavage *in vitro* and *in vivo* (9). While these findings unveil a new mechanism by which damaged podocytes can contribute to downstream tubular injury, additional research is needed to further validate the role of RARRES1 in kidney disease progression.

### Cytotoxicity of complement components and complement activation

Under proteinuric conditions, aberrantly filtered complement precursors enter the tubular lumen, where they can be activated on tubular membranes. This may be explained by the scant expression of complement inhibitors on the apical surface of tubular epithelial cells, which makes them vulnerable to complement-mediated cellular damage (10). *In vitro*, proximal tubular cells exposed to sublytic amounts of normal human serum activated the alternative pathway of complement, leading to the insertion of C5b-9 on cell surface and the generation of inflammatory mediators, including prostaglandin E2, interleukin (IL)-6, and tumor necrosis factor-α (TNF-α) (11). In mice with albumin-overload nephropathy, C3 deficiency attenuated both proteinuria and tubular damage (12). In the same model, the results of experiments with C3 deficient kidneys transplanted into wild-type mice and vice versa suggest that ultrafiltered C3 may be more critical for the development of tubulointerstitial injury than locally generated C3 (12).

Several lines of evidence suggest that properdin plays a critical role in tubular activation of the alternative complement pathway during proteinuria. In kidney biopsies from patients with proteinuric kidney diseases, properdin was detected on the brush border of the proximal tubules (13). Moreover, the presence of properdin in the urine of proteinuric patients associated with increased urinary excretion of sC5b-9, the soluble form of the terminal membrane attack complex, and with worse kidney function (14). *In vitro* experiments showed that properdin binding to the brush border was the rate-limiting step for tubular complement activation (13). Heparan sulfate expressed on tubular epithelium was identified as the ligand for properdin during proteinuria, working as a docking station for alternative pathway complement activation (15, 16). Factor H, one of the major fluid phase and cellular surface regulators of the alternative complement pathway, also binds to heparan sulfate, albeit at a different site from that of properdin (17), suggesting a delicate balance between complement activation and inhibition that can be skewed under proteinuric states. This hypothesis is supported by an *in vitro* study showing that protein overload reduced both heparan sulfate density and Factor H binding to proximal tubular cells, hence enhancing local complement activation (18).

Other aberrantly filtered plasma proteins, including plasminogen and urokinase-type plasminogen activator (uPA), can also facilitate tubular complement activation. *In vitro* studies have shown that the combination of plasminogen and uPA, but not either protein alone, activated C3 and C5 to the anaphylatoxins C3a and C5a, respectively, and this effect was inhibited by amiloride, an off-target inhibitor of uPA (19). In conditional podocin knockout mice with severe proteinuria, the elevated urinary excretion of C3a and C5a and kidney tissue expression of the inflammasome protein NOD-like receptor family, pyrin domain containing 3 (NLRP3) were significantly reduced by treatment with an anti-uPA monoclonal antibody, without altering proteinuria (19). These findings suggest that during proteinuria active plasmin produced by uPA-mediated cleavage of plasminogen contributes to intratubular generation of anaphylatoxins and downstream pro-inflammatory signals.

### Proteinuria-induced tubular cell apoptosis and tubular injury

Evidence from several *in vitro* and *in vivo* studies suggests that proteinuria causes tubular cell apoptosis, which is involved in the pathogenesis of tubular atrophy (20). In cultured proximal tubular cells, albumin overload activated both of the main pathways of apoptosis, that is the extrinsic—mediated by integral membrane death receptors belonging to the tumor necrosis factor superfamily—(21, 22) and the intrinsic—mediated by mitochondria—(23, 24) pathways. The contribution of the former was demonstrated by activation of Fas-Fas-associated protein with death domain-caspase-8 and death receptor 5-caspase-8 in cultured porcine and human proximal tubular cells, respectively, upon exposure to high albumin concentrations (21, 22). As for the intrinsic pathway, albumin was found to cause apoptosis of human proximal tubular cells by stimulating the translocation of the proapoptotic protein Bax to the mitochondrial membrane, followed by release of cytochrome c and activation of caspase-9 (23). This pathway was confirmed in rat proximal tubular cells, where activation of protein kinase C-δ was identified as an upstream event in albumin-triggered apoptosis (24). Consistently, protein kinase C-δ knockout mice showed attenuation of albumin overload-induced tubular cell apoptosis (24).

Proximal tubular cell apoptosis was reported to contribute to glomerular-tubule disconnection with the eventual formation of atubular glomeruli in several congenital and acquired kidney diseases, more frequently in tubulointerstitial disorders, including pyelonephritis and obstructive nephropathy, but also in disorders of glomerular origin, such as chronic glomerulonephritis and diabetic nephropathy (25, 26). The disconnection typically occurs at the site of the glomerular-tubule junction, which is especially vulnerable to injury. The formation of atubular glomeruli is thought to involve interstitial inflammation in the setting of tubulointerstitial diseases, and tubular toxicity of filtered proteins in the context of glomerular diseases (25, 26). Indeed, in rats with passive Heymann nephritis chronic treatment with an angiotensin-converting enzyme (ACE) inhibitor resulted in the prevention of both tubular atrophy and disconnection, possibly by reducing proteinuria-induced apoptosis of proximal tubular cells (27). In the same experimental model of proteinuric kidney disease, protection against glomerular-tubule disconnection was also achieved by chronic treatment with the sodium-potassium adenosine triphosphatase inhibitor ouabain, which reduced proximal tubular cell apoptosis by down-regulating Bax and up-regulating the antiapoptotic factor Bcl-xL (28). The signaling pathway whereby ouabain exerts its antiapoptotic effect appears to involve increased calcium release from endoplasmic reticulum stores, with subsequent activation of the nuclear factor-κB (NF-κB) p65 subunit, a transcriptional regulator of Bcl-xL (28, 29). Ouabain was also found to rescue cultured proximal tubular cells from high glucose-induced apoptosis by reducing mitochondrial depolarization and reactive oxygen species (ROS) generation (30). In another study, the use of intravital two-photon technology revealed that salt loading in Dahl salt-sensitive rats resulted in increased glomerular filtration and tubular reabsorption of albumin (31). This was followed by marked tubular damage, characterized by the presence of granular casts in the tubular lumen, apoptotic cell debris, dilation and necrosis of proximal tubular epithelial cells (31). Further experimental evidence on the tubular toxicity of filtered albumin came from the generation of a mouse model of Alport syndrome knockout for albumin (32). In fact, genetic deficiency of albumin in Alport mice resulted in reduced tubular cell apoptosis, tubulointerstitial injury, and glomerulosclerosis, ultimately slowing kidney disease progression (32).

Accumulating evidence indicates that during albumin overload, aberrantly filtered free fatty acids (also known as non-esterified fatty acids) bound to albumin are reabsorbed by the proximal tubule and contribute to cytotoxicity (33, 34). Since albumin has about seven fatty acid binding sites, under proteinuric conditions the concentrations of free fatty acids reaching the tubule lumen are considerably increased. Excessive fatty acids are taken up at the apical surface of proximal tubular cells mainly by the transmembrane fatty acid transporter-2 (FATP2) protein, and trigger apoptosis (35). Indeed, in mice with albumin-overload proteinuria, global knockout for FATP2 gene (*Slc27a2*) resulted in the mitigation of proximal tubular cell apoptosis and tubular atrophy compared to wild-type animals with albuminuria (35). Similarly, global knockout for *Slc27a2* in two mouse models of diabetic kidney disease led to attenuation of tubular atrophy, albuminuria and glomerular filtration rate decline compared with diabetic mice expressing FATP2 (36). Even though improvement of diabetic kidney disease could be due to the absence of proximal tubule FATP2-dependent fatty acid uptake, global *Slc27a2* knockout mice also exhibited remarkably reduced fasting glycemia, which could have conferred indirect kidney benefits (36).

### Involvement of endoplasmic reticulum stress in proteinuria-induced tubular cell apoptosis and tubular injury

Proteinuria can trigger tubular cell apoptosis also by inducing endoplasmic reticulum (ER) stress, a change in ER homeostasis due to the accumulation of unfolded or misfolded proteins, which leads to the activation of the unfolded protein response (UPR). This signaling transduction pathway imposes adaptive programs to restore protein processing functions or, in the event of prolonged stress, triggers apoptotic cell death (37). Insights into the involvement of ER stress in proteinuria-induced tubular cell apoptosis came from a study that combined *in vitro* and *in vivo* molecular and pharmacologic strategies. In particular, the exposure of tubular cells to albumin was found to rapidly drive intracellular events that induced calcium release from the ER (38). The albumin-induced ER calcium depletion stimulated activating transcription factor 4 (ATF4)-dependent overexpression of lipocalin 2 (LCN2), which in turn triggered apoptosis of tubular cells through ROS generation (38). Consistently, LCN2 protein expression was markedly increased in renal tubules of patients with proteinuric nephropathies and of proteinuric mice. Treatment with 4-phenylbutyric acid, a chemical chaperone already in clinical use as adjunctive therapy for urea cycle disorders, reduced ER-induced apoptosis and tubulointerstitial lesions, while improving kidney function, in proteinuric mice (38).

Reticulon (RTN)1A, an endoplasmic reticulum membrane protein, has also been implicated in albuminuria-induced tubular cell injury mediated by increased ER stress and kidney disease progression. In a murine model of albumin-overload kidney disease, tubular cell-specific knockdown of RTN1A blunted ER stress and apoptosis of tubular cells, and reduced kidney fibrosis (39). Consistently, specific overexpression of RTN1A in tubular epithelial cells exacerbated diabetic kidney disease in mice, as assessed by increased tubular injury, tubulointerstitial fibrosis and kidney function loss (40). Mechanistically, RTN1A overexpression worsened both ER stress and mitochondrial injury in tubular cells under diabetic conditions by facilitating ER-mitochondria interactions (40).

These findings are of relevance from a translational perspective since, besides 4-phenylbutyric acid, additional drugs approved for clinical use, albeit not for chronic kidney disease (CKD), are able to alleviate ER stress (e.g., sunitinib, approved for renal cell carcinoma, or tauroursodeoxycholic acid, approved for primary biliary cirrhosis) (37), making them attractive for repurposing in proteinuric kidney diseases.

### Involvement of autophagy impairment in proteinuria-induced tubular cell apoptosis and tubular injury

Similarly to ER stress, autophagy can lead to either cell survival or death depending on the degree and type of stress. This evolutionary conserved process involves lysosome-dependent degradation of cytoplasmic components for clearance and reuse (41). Recycling can be performed either non-selectively, as a cellular response to nutrient deprivation, or selectively to remove specific cargoes, including protein aggregates (aggrephagy), mitochondria (mitophagy), or lysosomes (lysophagy) (42). Basal autophagic activities in the kidney is required for cellular homeostasis (43). Loss of the basal autophagic response in the entire tubular system of adult mice due to inducible knockout for the autophagy-related gene *Atg5* resulted in kidney function impairment (44). However, distal tubule-specific *Atg5* deletion did not affect kidney function, pointing to the importance of the basal autophagic response in the proximal tubule to maintain functional integrity (44).

The exposure of cultured proximal tubular epithelial cells to albumin or protein overload led to autophagy activation as a protective mechanism against cellular injury (45, 46). Nevertheless, excessive albumin absorption and degradation impaired autophagy in proximal tubular cells through activation of the mammalian target of rapamycin (mTOR) pathway (47). Cell exposure to rapamycin, an inhibitor of mTOR, enhanced autophagy and attenuated albumin-induced proximal tubular cell apoptosis (45, 48). Instead, blocking autophagic degradation with chloroquine produced opposite effects (45). Similar findings were observed in proximal tubular cells of rats with high-grade proteinuria after treatment with rapamycin and chloroquine (45).

Persistent proteinuria can also compromise selective autophagy, with the ensuing intracellular build-up of damaged organelles, such as mitochondria, which trigger tubular injury. In cultured proximal tubular epithelial cells, albumin exposure led to reduced NIP3-like protein X (NIX)-mediated mitophagy, resulting in the accumulation of defective mitochondria that released ROS into the cytoplasm, thereby activating a mitochondrial-related cell apoptotic pathway (49). Similarly, mice with albumin overload-induced proteinuria exhibited reduced NIX-mediated mitophagy in renal tubules, along with increased mitochondrial fragmentation, tubular epithelial cell apoptosis, and kidney dysfunction (49). Overexpression of NIX attenuated albumin-induced tubular epithelial cell apoptosis both in culture and in proteinuric mice. Along this line, overexpression of Parkin in order to upregulate the phosphatase and tensin homolog (PTEN)-induced kinase1 (PINK1)/Parkin-dependent mitophagy pathway was found to mitigate albumin overload-induced mitochondrial dysfunction and apoptosis in cultured proximal tubular epithelial cells (50).

Together, data from *in vitro* and *in vivo* models suggest that in the initial phase of proteinuria autophagy may protect proximal tubular epithelial cells from injury. Nevertheless, chronic proteinuria likely exerts toxic effects on tubular cells by suppressing autophagic activities, thereby promoting tubular damage. While some autophagy-promoting strategies provided renal cytoprotective effects in animal models of proteinuric kidney diseases, evidence that such findings can be translated to the clinical setting is as yet absent.

### Proteinuria-induced phenotypic changes in tubular cells

Abnormally ultrafiltered proteins have been reported to promote proximal tubule phenotypic changes. One of these is cellular senescence, a state of irreversible growth arrest accompanied by the acquisition of a senescence-associated secretory phenotype, which entails the release of pro-inflammatory and pro-fibrotic factors. Cellular senescence has been documented in the tubular compartment of kidney biopsy specimens from diabetic patients with advanced diabetic kidney disease and even in those with milder proteinuria and more preserved glomerular filtration rate, suggesting that this process may occur early in the course of CKD (51). DNA damage secondary to oxidative stress, radiations or chemical agents is regarded as the main cause of cellular senescence (52). In cultured proximal tubular epithelial cells albumin overload was found to cause ROS overproduction, which induced DNA injury (21). The subsequent DNA damage response signaling resulted in cellular senescence, as assessed by increased protein expression of the markers p16, p21 and β-galactosidase, and the acquisition of a senescence-associated secretory phenotype, with overproduction of the pro-inflammatory cytokine IL-1β and the pro-fibrotic mediator transforming growth factor-β1 (TGF-β1) (21). A recent study showed that the senescence markers p21 and γ-H2AX were detected at higher levels in the proximal tubule of podocin-knockout mice, an experimental model of proteinuria, than in megalin-cubilin-podocin triple-knockout mice, as was the case for the mRNA expression of monocyte chemoattractant protein-1 (MCP-1) and TGF-β1 (53). These findings suggest that proteinuria can directly induce cellular senescence in proximal tubular cells by megalin/cubulin-mediated endocytosis of filtered proteins.

In recent years, the single-cell/nucleus RNA sequencing technology has been used to enhance our understanding of the pathophysiology of proteinuric kidney diseases in mice and humans. In particular, a study that combined single-nucleus RNA sequencing and intravital imaging to get insights into the tubular responses to glomerular proteinuria along the whole nephron in mice showed the initial extension of the protein reuptake capability from S1 to S2 segments of the proximal tubule by means of the transition of S2 cells into a S1/S2 hybrid cell state (54). This was followed by the appearance of injury and failed repair cell populations in the proximal tubule (54). Meanwhile, enhanced protein delivery to the distal tubule led to the dedifferentiation of the epithelium and reduced protein expression of solute transporters (54). In humans, single-cell RNA sequencing analyses of kidney biopsy specimens from CKD patients of different etiologies have shown changes in molecular signatures along with the severity of proteinuria. In IgA nephropathy, genes involved in leukocyte transendothelial migration, chemokine signaling, and type I interferon signaling pathways were upregulated in proximal tubular cells of patients with overt proteinuria compared to the group with lower-grade proteinuria (55). Similar findings were observed in primary membranous nephropathy, where genes participating in the regulation of inflammation and immune response were expressed at higher levels in most of the kidney parenchymal cells, including proximal tubular cells, of patients with nephrotic-range proteinuria compared to those with lower grade proteinuria (56).

## Interstitial inflammation and injury

A great deal of the proximal tubule phenotypic changes promoted by excessive reabsorption of ultrafiltered proteins can be ascribed to the activation of intracellular signaling pathways resulting in the production of inflammatory and pro-fibrotic cytokines. These substances are released into the interstitium, where they foster the recruitment of inflammatory cells and the activation of fibroblasts, thereby contributing to the development of progressive inflammation and fibrosis.

### Inflammation

Early *in vitro* studies have documented that plasma proteins, especially albumin, activated the NF-κB pathway in cultured proximal tubular cells leading to the production of pro-inflammatory molecules, such as MCP-1, regulated upon activation, normal T cell expressed and secreted (RANTES), IL-8, and fractalkine (57–60), which in turn fostered the recruitment of monocytes and macrophages in the tubulointerstitial space in rodents with albumin overload proteinuria (60, 61). More recent research has shown that albumin-induced tubulointerstitial inflammation involves activation of the NLRP3 inflammasome, a cytoplasmic multiprotein complex which integrates various danger signals into caspase-1 maturation, with the consequent processing and release of the proinflammatory cytokines IL-1β and IL-18 (62, 63). Studies *in vitro* and in a rat model of proteinuric nephropathy have suggested that excessive cubilin/megalin-mediated albumin uptake by proximal tubular cells overwhelmed lysosomal degradation activity leading to lysosomal rupture and release of hydrolases, especially cathepsin B, which trigger NLRP3 activation (62). Endoplasmic reticulum stress and mitochondrial dysfunction have also been identified as mechanisms by which albumin overload can induce NLRP3 activation in proximal tubular cells, with the eventual IL-1β and IL-18 production (64, 65). In turn, IL-1β from tubular epithelial cells of diabetic mice was found to promote the polarization of macrophages into a pro-inflammatory M1 phenotype characterized by high release of the pro-inflammatory cytokine IL-6 (66).

In addition to soluble cytokines, extracellular vesicles carrying functional cargoes, including exosomes and microvesicles, have emerged as critical mediators in the transmission of albumin-induced inflammatory signals from tubular epithelial cells to macrophages. *In vitro* and *in vivo* studies in 5/6 nephrectomized rats have documented that albumin overload can induce tubular epithelial cells to release exosomes enriched in CC-chemokine ligand 2 (CCL2, also referred to as MCP-1) mRNA, which are delivered to interstitial macrophages, leading to their activation (67). Moreover, tail-vein injection of extracellular vesicles secreted from albumin-stimulated tubular epithelial cells into diabetic mice drove polarization of kidney macrophages toward the M1 pro-inflammatory phenotype by the delivery of miR-199a-5p, thereby accelerating diabetic kidney disease progression (68). Injection of these extracellular vesicles in diabetic mice was also found to switch the metabolism of kidney macrophages toward glycolysis by stabilizing hypoxia-inducible factor-1α, a transcription factor that upregulates glycolysis-related gene, an effect that was associated with increased renal inflammation and fibrosis (69).

### Adaptive immune response

Inflammatory mediators produced by proximal tubular cells in response to protein overload can act as danger signals that prompt kidney dendritic cells to initiate an immune response against normally ignored self-antigens, such as albumin peptides. *In vitro*, the exposure of rat proximal tubular cells to high concentrations of autologous albumin led to proteolytic cleavage and release of the N-terminal 24-residue fragment of albumin (ALB_1 − 24_), which was further processed by dendritic cells into antigenic peptides that were loaded on MHC class I for presentation to and activation of CD8^+^ T cells (70). In rats made proteinuric by 5/6 nephrectomy, dendritic cells infiltrated into the renal parenchyma, and subsequently migrated to the kidney draining lymph nodes. Dendritic cells harvested from renal lymph nodes and pulsed with the albumin peptide ALB_1 − 24_ activated syngeneic CD8^+^ T cells in primary culture (70). These findings indicate that the concerted action of proximal tubular cells and dendritic cells can generate antigenic peptides from albumin, which trigger an autoimmune response upon kidney injury. Besides helping dendritic cells in their role of antigen-presenting cells, proximal tubular cells can act themselves as antigen-presenting cells and stimulate antigen-specific CD4^+^ and CD8^+^ T cell activation *in vitro* (71, 72). Whether they may be able to present albumin-derived peptides to CD8^+^ T cells is an intriguing possibility that remains to be tested.

### Fibrosis

**Figure 2:**
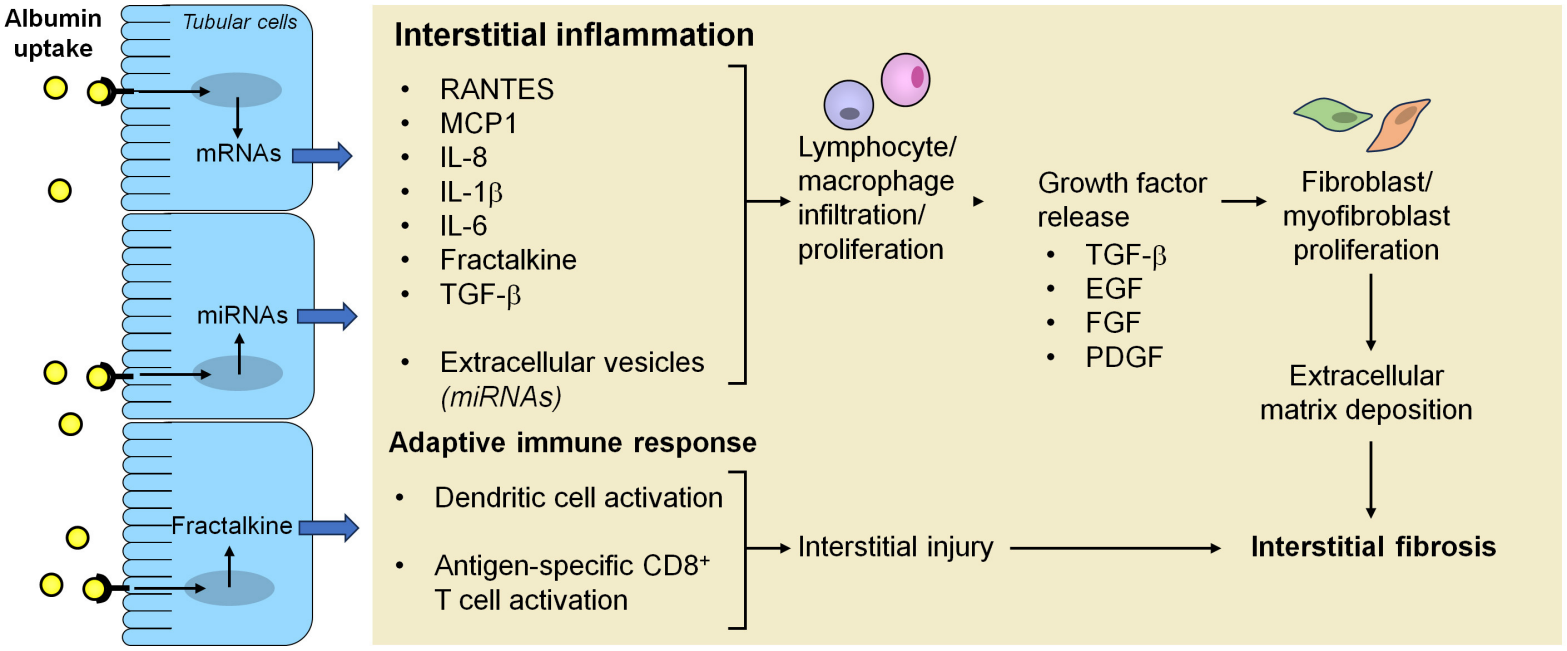
Mechanisms underlying the activation of inflammation, adaptive immunity and fibrosis in the interstitium after proximal tubular cell uptake of ultrafiltered proteins. Albumin overload of proximal tubular cells as a consequence of the breakdown of the glomerular filtration barrier integrity activates intracellular signals that lead to increased production of inflammatory mediators and growth factors, which can be released both as soluble factors and in extracellular vesicles, resulting in progressive inflammation and fibrosis. Albumin reabsorbed from proximal tubular cells can also undergo proteolytic cleavage into fragments that are further processed by dendritic cells into antigenic peptides which are loaded into MHC class I for presentation to and activation of CD8^+^ T cells, eventually contributing to interstitial injury. EGF, epidermal growth factor; FGF, fibroblast growth factor; IL-1β, interleukin-1β; IL-6, interleukin-6; IL-8, interleukin-8; MCP-1, monocyte chemoattractant protein-1; miRNA, microRNA; PDGF, platelet-derived growth factor; RANTES, regulated upon activation, normal T cell expressed and secreted; TGF-β, transforming growth factor-β.

Kidney fibrosis, the final common sequelae of proteinuric chronic kidney diseases, is characterized by excessive deposition of extracellular matrix in the interstitium. Activated myofibroblasts, a specialized population of fibroblasts, are known to fuel this process by secreting matrix components, such as collagen and fibronectin, but their origin has long been debated. Tubular epithelial cells were initially thought to be a key source of myofibroblasts through a mechanism called epithelial-to-mesenchymal transition (EMT). Nevertheless, this hypothesis was primarily supported by colocalization of mesenchymal and tubular epithelial markers (73). Later studies using lineage tracing strategies have shown that very few tubular epithelial cells, if any, are able to cross the basement membrane and become myofibroblasts (74, 75). Consistently, a recent single-cell RNA sequencing analysis of healthy and fibrotic human kidneys identified distinct populations of pericytes and fibroblasts as the major cellular source of myofibroblasts, whereas tubular epithelial cells had a minor role (76). Nonetheless, *in vivo* evidence from mice with tubular epithelial cell-specific genetic ablation or overexpression of transcriptional regulators of the EMT program, *Snai1* or *Twist*, supports the contribution of partial EMT to kidney fibrosis (74, 75). During this process, injured tubular epithelial cells acquire mesenchymal-like characteristics while remaining within the basement membrane and secrete pro-fibrotic factors, such as TGF-β, which drive the differentiation of interstitial fibroblasts into myofibroblasts and release cytokines that recruit macrophages (74, 75, 77, 78) (Figure 2). Accordingly, inhibition of SNAI1 in mice after induction of unilateral ureteral obstruction resulted in a substantial reduction of renal fibrosis and inflammation (75). These observations are in line with an earlier study in rats with remnant kidneys showing that abnormal uptake of ultrafiltered proteins by proximal tubular cells increased gene expression of TGF-β1, thereby leading to peritubular accumulation of α-smooth muscle actin-positive myofibroblasts, which co-localized with macrophages (79). Damaged tubular epithelial cells can trigger fibroblast-to-myofibroblast differentiation also by reactivation of developmental signaling proteins. Among these, Wnt9a was barely detectable in normal adult kidneys, but upregulated in the tubular epithelium of patients with proteinuric kidney diseases, including IgA nephropathy, membranous nephropathy and diabetic nephropathy (80). *In vitro* evidence showed that Wnt9a acted on proximal tubular cells increasing the expression of senescence proteins and the production of TGF-β1, which promoted proliferation of kidney fibroblasts and their conversion to myofibroblasts (80). TGF-β1 also stimulated the expression of Wnt9a in fibroblasts, thereby creating a reciprocal activation cycle between senescent tubular cells and activated fibroblasts (80). Similar to Wnt9a, sonic hedgehog was upregulated in the tubular epithelium of patients with proteinuric kidney diseases, such as IgA nephropathy, membranous nephropathy and focal segmental glomerulosclerosis (81), as it was in animal models of CKD induced by ischemia/reperfusion injury, adriamycin, renal mass ablation, or diabetes mellitus (81, 82). Studies *in vitro* and in experimental unilateral ureteral obstruction, ischemia/reperfusion injury and diabetic kidney disease suggest that proximal tubular cells secrete sonic hedgehog, both as soluble factor and released in extracellular vesicles, which stimulated interstitial fibroblast activation and proliferation, eventually leading to kidney fibrosis (81–83).

Injured tubular epithelial cells can promote renal fibrosis also through the production of microRNAs (miRNAs). In this regard, tubular expression of miR-214 was found to be higher in kidney biopsy specimens from CKD patients than from healthy controls, and directly correlated with the degree of proteinuria and renal fibrosis (84). Likewise, miR-214 was upregulated in tubular cells of mice with CKD induced by unilateral ureteral obstruction, ischemia/reperfusion injury, or albumin overload (84). The specific proximal tubular deficiency of miR-214 blunted inflammation and mitochondrial damage in these CKD models, with renal interstitial fibrosis being also alleviated in mice under unilateral ureteral obstruction or post-ischemia-reperfusion conditions (84). Mechanistically, miR-214 downregulated the mitochondrial genes mt-*Nd4l* and mt-*Nd6* in proximal tubular cells, thereby promoting progression of CKD due to different etiologies by disrupting mitochondrial oxidative phosphorylation (84). Another miRNA, miR-184, was found to be upregulated in renal tubules from Zucker diabetic fatty rats with overt nephropathy, and associated with collagen accumulation and reduced expression of lipid phosphate phosphatase 3 (LPP3), an enzyme involved in the clearance of the pro-fibrotic mediator lysophosphatidic acid (85). In cultured proximal tubular cells, albumin acted as a potent inducer of miR-184 which, in turn, promoted LPP3 downregulation and overexpression of plasminogen activator inhibitor-1, a serine protease inhibitor that attenuates fibrinolysis and promotes extracellular matrix accumulation (85). In Zucker diabetic fatty rats with established kidney disease, ACE inhibitor treatment reduced albuminuria and limited renal miR-184, with tubular LPP3 preservation and tubulointerstitial fibrosis attenuation (85). Together, these findings suggest that miR-184 works as a downstream effector of albuminuria, promoting kidney fibrosis in rats with diabetic nephropathy (85).

In addition to cellular components, soluble factors and extracellular vesicles, the extracellular matrix network, whose qualitative and quantitative composition varies over the course of CKD, actively contributes to the development of kidney fibrosis (86). A recent proteomic profiling study of decellularized kidney extracellular matrix scaffolds from healthy and CKD mice revealed that glutathione peroxidase 3 (GPX3), an antioxidant enzyme mainly secreted by proximal tubular cells, was downregulated in the fibrotic kidney (87). Knockdown of GPX3 worsened renal fibrotic lesions in a mouse model of unilateral ureteral obstruction through the generation of an oxidatively stressed milieu by enhancing the nicotinamide adenine dinucleotide phosphate oxidase 2/ROS/p38 mitogen-activated protein kinase signaling cascade (87). Although these findings were observed in an experimental model of kidney damage unrelated to proteinuria, the evidence that GPX3 is downregulated in renal biopsy samples from patients with membranous nephropathy and IgA nephropathy (87), suggests that such pathway can be altered also in the setting of proteinuric kidney diseases (88).

## Tubulointerstitial repair

The kidney has endogenous repair mechanisms that are activated in response to injury. In particular, damaged tubular cells are an important source of M2 macrophage polarization factors, such as macrophage colony-stimulating factor, granulocyte-macrophage colony stimulating factor, and Nectrin 1 (89). In turn, M2 macrophage-derived Wnt7b and chitinase-like protein BRP-39 promote tubular repair in mice with kidney ischemia-reperfusion injury by limiting apoptosis (90, 91). In the same model, the macrophage-derived protein apoptosis inhibitor of macrophage (AIM) on intraluminal debris was found to interact with kidney injury molecule-1 expressed by injured tubular epithelial cells to induce the clearance of the dead-cell debris, thereby facilitating recovery from acute kidney injury (92). Manipulations of macrophages toward an M2 phenotype have been explored as a treatment of proteinuric kidney diseases. In this regard, *in vivo* induction of M2 macrophages by injection of IL-25 in mice with adriamycin nephropathy resulted in reduced glomerulosclerosis, tubular atrophy, interstitial volume and urinary protein excretion (93). Similar protective effects against adriamycin nephropathy were documented by transfusion of macrophages isolated from mice undergoing peritoneal dialysis and polarized to M2 phenotype by stimulation with IL-4/IL-13 (94).

In recent years IL-11, a pro-inflammatory cytokine belonging to the IL-6 family, has emerged as a therapeutic target for fibrosis and endogenous repair in diverse organs and tissues, including the kidney (95). Human tubular epithelial cells were found to act both as a target and source of IL-11. They express IL-11 receptor subunit alpha (IL-11RA) and, in response to a variety of stimuli, such as TGF-β and angiotensin II, release IL-11, which in turn promotes SNAI1 upregulation in tubular epithelial cells, resulting in their exit from the cell cycle and conversion to a dysfunctional partial EMT phenotype (96). Notably, administration of an anti-IL-11 in mice with established CKD induced by folic acid led to reduced renal fibrosis and inflammation, while restoring kidney parenchymal mass and function (96). Mechanistically, inhibition of IL-11 reversed SNAI1-driven partial EMT and induced stalled tubular epithelial cells to re-enter the cell cycle and proliferate, resulting in kidney repair and regeneration (96). IL-11 signaling was also studied in mice knockout for the type IV collagen α3 chain (*Col4a3*^−/−^), an experimental model of Alport syndrome (97). In this context IL-11 was found to be upregulated in the tubular epithelium, an effect accompanied by increased expression of SNAI1—which indicates a partial EMT state -, tubular damage, kidney fibrosis, and early mortality (97). Treatment of *Col4a3*^−/−^mice with anti-IL-11 antibody from 6 weeks of age, a time point following proteinuria development and renal IL-11 upregulation, mitigated the above effects (97). In particular, the median lifespan of *Col4a3*^−/−^mice was extended by 29 days (44%) with anti-IL-11 antibody, 14 days (22%) with an ACE inhibitor, and 62 days (99%) with ACE inhibitor and anti-IL-11 antibody combination therapy (97). Thus, blockade of IL-11 appears to exert independent and additive therapeutic benefits relative to ACE inhibition in *Col4a3*^−/−^mice. Moreover, the observation that urinary IL-11 excretion directly correlated with proteinuria in patients with IgA nephropathy and lupus nephritis (98), raises the possibility that IL-11 signaling may be of relevance also in other proteinuric chronic kidney diseases. Interesting is also that treatment of middle-age mice with the aforementioned anti-IL-11 antibody has recently been reported to boost metabolism, reduce frailty, and prolong the median lifespan of male and female mice by 22.5% and 25%, respectively (99). Together, these observations, coupled with anti-IL-11/anti-IL-11RA antibodies currently being under early stage clinical development for idiopathic pulmonary diseases (95), may provide the background for future clinical trials testing such novel therapeutic class of drugs in progressive CKD added on top of standard of care with optimally dosed renin-angiotensin-aldosterone system blockers and sodium-glucose cotransporter-2 inhibitors.

## Conclusions

A wealth of studies in experimental models of proteinuric kidney diseases have expanded our understanding of how the load of proteins that pass through the breached glomerular filtration barrier eventually promote tubular damage, interstitial inflammation, and fibrosis. The main mechanisms include intratubular complement activation, excessive proximal tubular reuptake of albumin/proteins, which can lead to either apoptosis or the acquisition of a pro-inflammatory phenotype. All of these processes could be potential targets for therapeutic interventions. A complementary approach may involve fostering the recently identified endogenous tubulointerstitial repair mechanisms in the setting of established CKD. Moreover, the continuous advancements in single-cell and spatial multi-omics technologies are expected to uncover novel mechanistic insights into the pathogenesis of proteinuric kidney diseases in the near future, with the ultimate goal to develop tailored therapeutic strategies for CKD patients.
